# Supplementary material for: Laboratory culture of the California Sea Firefly Vargula tsujii (Ostracoda: Cypridinidae): Developing a model system for the evolution of marine bioluminescence
Source: Sci Rep. 2020 Jun 26;10:10443. doi: 10.1038/s41598-020-67209-w (PMC7320024; doi:10.1038/s41598-020-67209-w)
Supplement: Supplementary file 1 — Supplemenatry information. [file 41598_2020_67209_MOESM1_ESM.zip › Supplementary figures.docx]

Running title: Complete life cycle of the bioluminescent California Sea Firefly

**Supplementary Figures: Laboratory culture of the California Sea Firefly *Vargula tsujii* (Ostracoda: Cypridinidae): Developing a model system for the evolution of marine bioluminescence**

Jessica A. Goodheart^1,2^, Geetanjali Minsky^1^, Mira N. Brynjegard-Bialik^1^, Michael S. Drummond^1^, J. David Munoz^3^, Timothy R. Fallon^2,4,5^, Darrin T. Schultz^6,7^, Jing-Ke Weng^4,5^, Elizabeth Torres^3^ & Todd H. Oakley*^1^

^1^ Department of Ecology, Evolution, and Marine Biology, University of California, Santa Barbara, Santa Barbara, CA 93106

^2^ *Current address:* Scripps Institution of Oceanography, University of California, San Diego, La Jolla, CA 92093, USA

^3^ Department of Biological Sciences, California State University, Los Angeles, CA 90032-8201, USA

^4^ Whitehead Institute for Biomedical Research, Cambridge, MA, 02142, USA

^5^ Department of Biology, Massachusetts Institute of Technology, Cambridge, MA 02142, USA

^6^ Monterey Bay Aquarium Research Institute, Moss Landing, CA 95060, USA

^7^ Department of Biomolecular Engineering and Bioinformatics, University of California, Santa Cruz, Santa Cruz, CA 96060, USA

*Corresponding author: oakley@ucsb.edu

**Figure S1.** Traversal of the cleaned SPAdes *Vargula tsujii* mitochondrial genome assembly graph nodes plotted by Bandage ^57^ (gray) (v.0.8.1; <https://github.com/rrwick/Bandage>). The single circularizing path representing the collapsed duplicate control regions are shown in blue. The black stroke shows the path traversed through the assembly graph followed to produce the circularized mtDNA FASTA file with two control region, matching the structure of the V. hilgendorfii mtDNA (Ogoh and Ohmiya 2004).

**Figure S2.** Results for our *Vargula tsujii* 16S DAPC model test to determine the number of clusters favored via comparison of Bayesian Information Criterion (BIC) values.

**Figure S3.** Results for our *Vargula tsujii* COI DAPC model test to determine the number of clusters favored via comparison of Bayesian Information Criterion (BIC) values.

**Figure S4.** Cluster membership based on our *Vargula tsujii* 16S DAPC analysis compared with the original population designation for each specimen. Overall, the clusters are not representative of any population-level structure.

**Figure S5.** Cluster membership based on our 16S *Vargula tsujii* DAPC analysis compared with the original population designation for each specimen. Overall, the clusters are not representative of any population-level structure.

**Figure S6.** Exploded diagram of custom acrylic aquarium for rearing *Vargula tsujii*. A photo of thisbuilt aquarium is in the mainin main text, Figure 2.  A. Plumbing components include 1. PVC pipe 1.5 inch diameter. 2. PVC socket ball valve to control the rate of outflow 3. PVC 1.5 inch elbows 4. PVC elbow with one side threaded to screw into 5. threaded bulkhead to span bottom of aquarium for drainage 6. float switch valve to control water level (not shown on Figure 2 of main text). B. Acrylic panels of ¼ inch thickness welded together with solvent include 7. Two panels of dimension 16.5 x 13.25 inches that form the long sides of the aquarium 8. Two panels of dimension 8 x 10.75 inches that form the short sides of the aquarium. One of these panels has a hole drilled for the water inflow and float switch 9. One panel of 16.5 x 7.5 inches to form the bottom of the aquarium, including a hole drilled for the bulkhead 10. One panel of 16.5 x 7.5 inches to forms a false bottom for the aquarium similar to the bottom of an undergravel aquarium filter. We drilled small holes throughout to allow water but not sand to flow through. (Not shown on Figure 2). In practice, we welded legs on the false bottom to allow it to stand on the true bottom of the aquarium above the height of the bulkhead drain.

**Figure S7.** Images showing the rubber gaskets for the *Vargula tsujii* custom aquaria. At left is a top view of aquarium before adding gravel and sand. We added 1/8" nitrile to the false bottom, fastening with plastic screws, washers, and nuts through the nitrile and false bottom. The nitrile hangs approximately 1 cm over the edge of the false bottom, forming a seal with the sides of the aquarium so that sand, detritus, and ostracods will not fall past the false bottom. At right is an aquarium in use. The black nitrile is visible with sand above. We now place a layer of aquarium gravel (approximately 1.5 cm in size) directly on the false bottom, and place aragonite sand above the gravel.

**Figure S8.** Small aquarium (“condo”) setup using an inverted 500 ml bottle. a. Seawater feed through a hose into reservoir tank. b. Inverted bottle with hole in bottom and tube piercing septum cap c. Siphon to bring water to condo (note in this staged example for photo, reservoir tank level is higher than condo, so water would flow out the top of the condo) d. Reservoir tank receives water from inflow and overflows e. Stand to hold condos f. Outflow tube using ¼ inch aquarium tubing, raised to set water level.
